# Pointing over gaze: how saliency, proximity, and context shape spatial attention to embodied cues

**DOI:** 10.1007/s00426-026-02250-4

**Published:** 2026-02-07

**Authors:** Wieske van Zoest, Adam Higgins

**Affiliations:** School of Psychology Edgbaston, University of Birmingham, Edgbaston, Birmingham, B15 2TT UK

**Keywords:** Attention, Context, Social cues, Gaze cue, Pointing cue, Mental attribution

## Abstract

Recent work suggests that the pointing hand on an outstretched arm is possibly a more powerful cue than the gaze-cue, suggesting these embodied social cues are not equal. The aim of this study is to investigate differences between gaze- and pointing-cue, looking specifically at saliency, spatial proximity, and trial context. A cartoon figure was used to present four types of cues: (1) a gaze-cue, (2) a peripheral pointing cue on an outstretched arm, (3) a central pointing cue presented over the torso of the body, and (4) a flower cue matched for low-level features to the peripheral pointing cue. Validity was non-predictive. To test the impact of trial context on the impact of the cues, different cue types were presented randomly within blocks (Experiment 1) or tested in separate blocks (Experiment 2). In Experiment 3, gaze and gesture cues were intermixed within blocks, but the number of gaze-cue trials was balanced with the pointing cues, ensuring that the gaze-cue occurred equally often as the gesture cues. The results showed that the pointing cue was much more effective in directing attention than the gaze-cue, especially when cues could not be predicted (Experiment 1 and 3). Blocked conditions (Experiment 2) yielded more effective cue-effects compared to mixed conditions (Experiment 1) and even yielded reliable cue effects for stimuli without intuitive directional meaning (i.e., flower cue). Across all three experiments, the results showed that the impact of the pointing cue could not be explained by low-level salience or spatial proximity to the target. Trial context affected the effectiveness of the cues, suggesting that spatial cueing is shaped by expectations; however, the advantage of the pointing cue over the gaze cue emerged independently of trial context. Together, these results challenge the idea that embodied cues influence attention uniformly, revealing systematic variation in effectiveness, with the pointing cue especially robust.

Gaze direction and pointing gestures are powerful cues for orienting attention. Stimuli appearing at locations that are gazed at (e.g., Bayliss & Tipper, 2006; Driver et al., 1999; Friesen & Kingstone, 2003; McKay et al., 2021) or pointed to are responded to more quickly than stimuli at uncued locations (Lu & van Zoest, 2023; Sato et al., 2010). Unlike symbolic, non-social cues such as arrows, these embodied social cues are often assumed to engage mental state attribution processes (e.g., Morgan et al., 2018; Nuku & Bekkering, 2008; Teufel et al., 2010; Wiese et al., 2013). However, despite this theoretical distinction, many studies report that arrow cues produce attentional effects comparable to those of gaze cues (e.g., Chacón-Candia et al., 2023; Tipples, 2002; however see, Schmitz et al., 2024). In contrast, growing evidence suggests that pointing cues may exert a stronger influence on attention than gaze cues (e.g., Burton et al., 2009; Hermens et al., 2017; Lu & van Zoest, 2023). The present study aims to identify factors that may account for differences in the efficacy of embodied social cues, focusing specifically on gaze and pointing. To this end, we examine the roles of cue saliency, spatial proximity, and trial context in modulating attentional orienting.

The majority of studies that directly compared the pointing cue and the gaze cue have used separate images of a hand and face to test the cue effect (Burton et al., 2009; Sato et al., 2010; Hermens et al., 2017; however, see Langton & Bruce, 1999; Materna et al., 2008). For example, Sato et al. (2010) presented participants with a photographic stimulus of (1) a central face with averted eyes left or right, or (2) a central hand pointing left or right, or (3) a schematic arrow pointing left or right. The results revealed an almost identical cueing effect for gaze, pointing, or non-social arrow cues, and suggest a common psychological mechanism for automatic attentional shift triggered by gaze, gestures, and symbols. Hermens et al. (2017) similarly used isolated pictures of a face and hand; however, they presented these stimuli in the periphery. Participants were instructed to respond to the cue direction of one specific assigned cue (either the eyes, face, hand, or head), ignoring the direction cued by a second cue that could be congruent or incongruent to the target cue. In contrast to Sato et al. (2010), the results of Hermens et al. (2017) showed the hand and head cue were a more powerful distractor cues than the face and eyes. The authors concluded that cues with a distinct outline, like the pointing cue, are more competitive in conflicting situations than cues without a distinct outline, like the gaze-cue.

Different from studies that use separate pictures of and hand or face to investigate and compare the pointing and gaze-effect, Lu and van Zoest (2023) recently developed a cartoon figure in which the pointing cue and gaze-cue are presented in one personified cartoon picture. The benefit here is that the pointing and gaze-cue can be manipulated in the same figure, and approximate proportional and positional differences inherent to the cues. That is the eyes are typically smaller than an outstretched pointing arm and hand; the eyes are central to the face, while a pointing arm is peripheral to the faces and eyes. The cartoon figure was used to manipulate the direction of the pointing cue, the gaze-cues, or both. Across all experiments, the cues were always completely non-predictive of target location. The results of the first experiment showed that the impact of the pointing cue was much larger than the gaze-cue (Lu & van Zoest, 2023). The results furthermore showed there was no added benefit of an aligned gaze-cue to pointing cue performance; also, when the gaze cue provided conflicting information, it did not reduce the impact of the pointing cue. In other words, the pointing cue provided an equally strong cueing effect, regardless of where the gaze was directed, suggesting that the pointing cue dominated biases in attention.

There are at least two possibilities that could explain why pointing cues are perceived as more powerful in directing attention than gaze (Hermens et al., 2017; Lu & van Zoest, 2023). First, the distance between actual features of the cue and response target can influence the relative impact of the gaze- and pointing cue. Because the pointing hand extends from the body, the hand is closer to the target compared to the central gaze-cue and this could facilitate target processing. Also, given that pointing cues typically extend from the body, it may represent a spatially more precise cue compared to gaze cues. Previous research has found that the spatial specificity (the size of the area cued) of gaze cues was quite poor and covered the entirety of the hemifield unless reference objects in the periphery provided contextual information (Wiese et al., 2013). Compared to gaze-cues, pointing cue may provide more spatially specific guidance even in the absence of contextual placeholders.

Second, pointing cues may be more effective cue due to an increased salience of the lateralized arm gesture, as opposed to the centralized subtle gaze-cue. Hermens and colleagues (2017; 2018) found that when cues were presented in extrafoveal vision, pointing hands lead to greater validity effects compared to gaze cues presented in extrafoveal vision. It was suggested that directionality of the pointing cue is easier to distinguish because it has a clear outline, compared to the directionality of the gaze-cue, which is embedded in the face. The enhanced response to the pointing cue was found in adults (Hermens et al., 2017) as well as children as young as three years old (Gregory et al., 2016). The fact that the cued direction of the pointing hand can be processed even when not fixated, compared with eye gaze cues which must first be processed through the fovea (central area of fixation), might help to explain why attention to cued targets is more effective in pointing cues than gaze cues. In other words, the pointing cue might be more salient in terms of low-level features, explaining why it is more strongly draws and directs attention.

The distance-hypothesis and saliency-hypothesis are not mutually exclusive. The peripheral pointing cue with explicit outline is both more salient and closer to the target than the central gaze-cue. These two factors together might explain the observed interaction showing that pointing cues are more influential than the gaze-cue (Lu & van Zoest, 2023). While target proximity and stimuli-saliency are difficult to disentangle, comparisons across conditions can help clarify their respective contributions to the observed effects.

To investigate the role of (1) spatial distance to the target and, (2) low-level stimulus salience of the pointing cue, two novel cues were developed to test the influence of these possible factors. A pointing cue was created in which the hand-cue was presented centrally crossed over the torso of the figure (see Fig. 1C). Presenting the pointing cue centrally changes two important things. First, it reduces low-level stimulus-driven differences compared to when the cue is presented on outstretched arm in the periphery, and secondly, is reduces the distance from the cue to the target. A second ‘pointing’ cue was developed in which the figure was holding a flower (see Fig. 1D), This figure was matched in low-level features to the peripheral pointing cue but did not involve any directionality. The flower-cue was similar both in saliency and cue-target distance to the peripheral pointing cue. In addition to these two novel pointing cues, a gaze-cue only condition (see Fig. 1A) was presented as well the as a ‘standard’ pointing cue condition with outstretched arm (peripheral pointing cue, Fig. 1B) used in previous research (Lu & van Zoest, 2023).

**Fig. 1:**
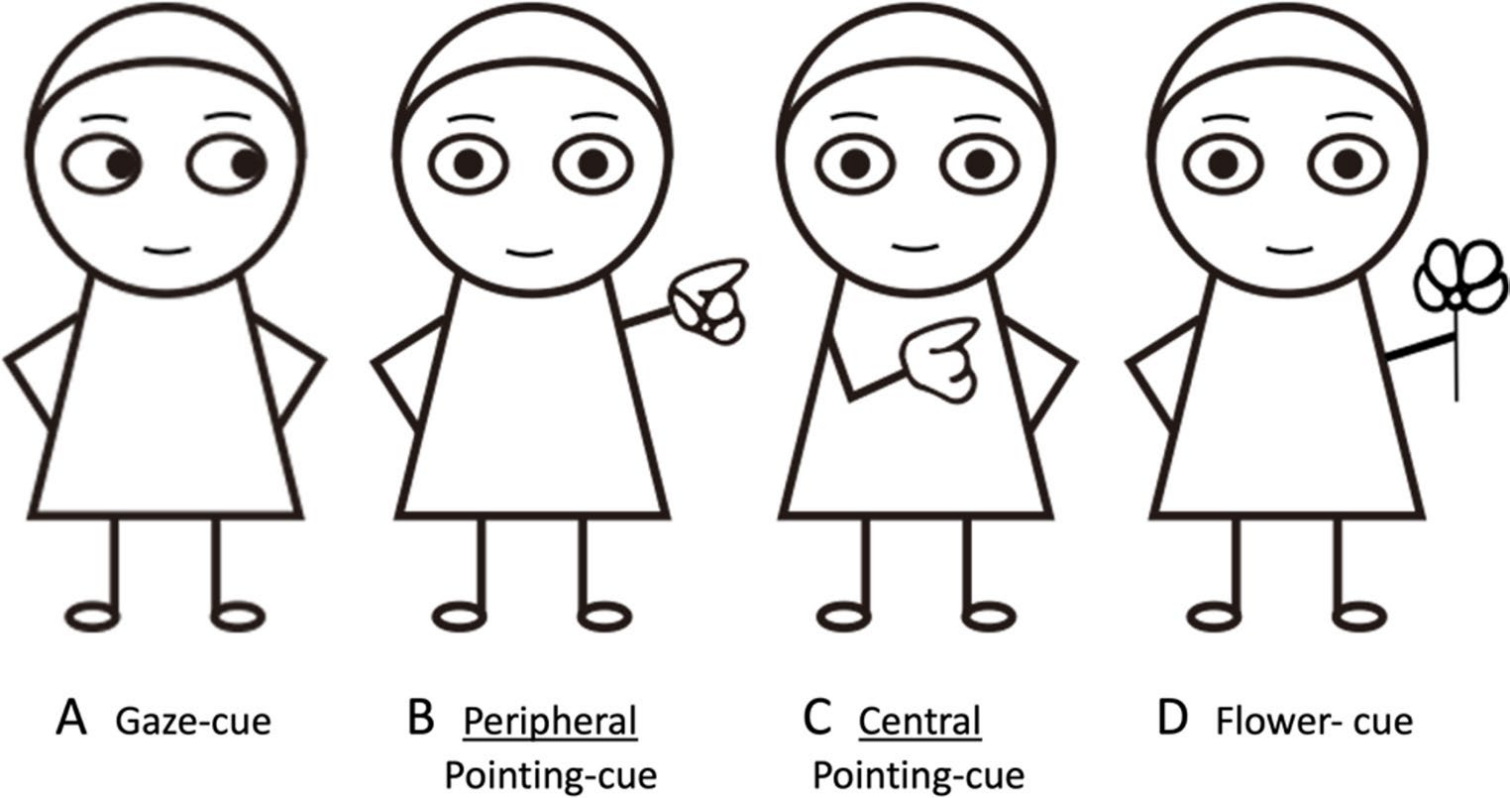
Note. Stimuli representing the four cue types, all cueing the right side of space

The first experiment aims to test at least three hypotheses. First, based on previous research (Lu & van Zoest, 2023), we expect to find a greater cue effect for the peripheral pointing cue (Fig. 1B) compared to the gaze cue (Fig. 1A). Second, if saliency and distance to the target of the pointing cue are critically important, we expect to find more effective cueing in the peripheral pointing cue (Fig. 1B) condition which is more salient and closer to the target compared to the central pointing cue (Fig. 1C) condition. If we find similar performance across both the central and peripheral pointing cue, this would suggest the pointing hand is uniquely effective in drawing attention independent of the saliency and distance to the target (see also, Ariga & Watanabe, 2009). Third, if low-level salience and distance to the target determine the strength of the pointing cue independent of intended directionality, we predict that the flower cue (Fig. 1D) should be equally strong as the peripheral pointing cue (Fig. 1B). If we find a distinctive benefit of the peripheral pointing cue over the flower cue, this would suggests the pointing hand is uniquely effective in drawing attention, independent of saliency or distance to the target (Ariga & Watanabe, 2009).

## Transparency and Openness

Power analyses were used to motivate sample sizes for each experiment and described in the “Participants” section for each experiment. Power analyses were performed with G*Power software v3.1 with α of 0.05 (Faul et al., 2007). Data exclusion parameters are reported; age and gender demographics are provided for each experiment. Participants were recruited from the United Kingdom only, and no other demographic information was considered. None of the experiments were preregistered. Data analysis was conducted using JASP (JASP Team, 2024). Data and stimuli materials are available at https://osf.io/ftrmc/.

## Experiment 1

### Method

#### Participants

A sample of United Kingdom residents was recruited on Prolific (https://www.prolific.co/). Inclusion criteria required a minimum of 70% Prolific approval rating. Informed consent of each participant was obtained prior to the study; the study was approved by the university Ethical Committee (protocol ERN-19–0260) and was conducted in accordance with the principles expressed in the Declaration of Helsinki. Thirty-four participants (11 male, 18 female, five undeclared) completed the study, their ages ranged from 22 to 65 years (*M* = 37.14, *SD* = 11.42). Participants were paid for their participation.

Sample size was decided based on previous study that combined gaze- and pointing cues (Experiment 1, Lu & van Zoest, 2023, *n* = 32). In this work the interaction between cue-type and validity had an effect size of η²= 0.42. To obtain the statistical power of 0.95 (Anderson et al., 2017), the current investigation required minimum sample size of 14 participants (alpha = 0.05). The current sample size of 34 was sufficient to evaluate the effect.

#### Apparatus

The experiment was created in OpenSesame (Mathot et al., 2012) and run online in OpenSesame extension, OSWeb (https://osdoc.cogsci.nl/3.3/manual/osweb/osweb/) and JATOS (http://www.jatos.org/) for data collection (Lange et al., 2015). The study link was provided on completion of an information and consent form on Qualtrics (https://www.qualtrics.com/uk/).

#### Stimuli

Cues were adapted from Lu and van Zoest (2023) and presented in Fig. 1. Height was identical for all cues. Width of cues was not equal, as the peripheral pointing cue and flower cues extended horizontally further than central pointing cue and gaze cues. Blank space of equal length to the outstretched arm was added to the opposite side of these cues to ensure the centre of the image was the same for all cues.

#### Design

Experiment 1 contained two within-subject factors: cue-type and validity. The independent variable cue-type had four levels: (1) gaze-cue, (2) peripheral pointing cue, (3) central pointing cue, and (4) flower-cue. See Fig. 1. Cues probed either the left or right side of the display and were not predictive of target location (i.e., 50% likely to point to the target). The independent variable validity was determined by the combination of target position (on the left or right) and cue direction (left or right): A valid trial occurred where the target was presented on the side prompted by the cued. An invalid condition was one where the target was presented on the side opposite to the one prompted by the cue. Conditions were presented one at a time, in random order. The target letter was either a letter F or H and presented on the left or right of the central character. A distractor letter X was presented opposite to the target location.

#### Procedure

**Fig. 2:**
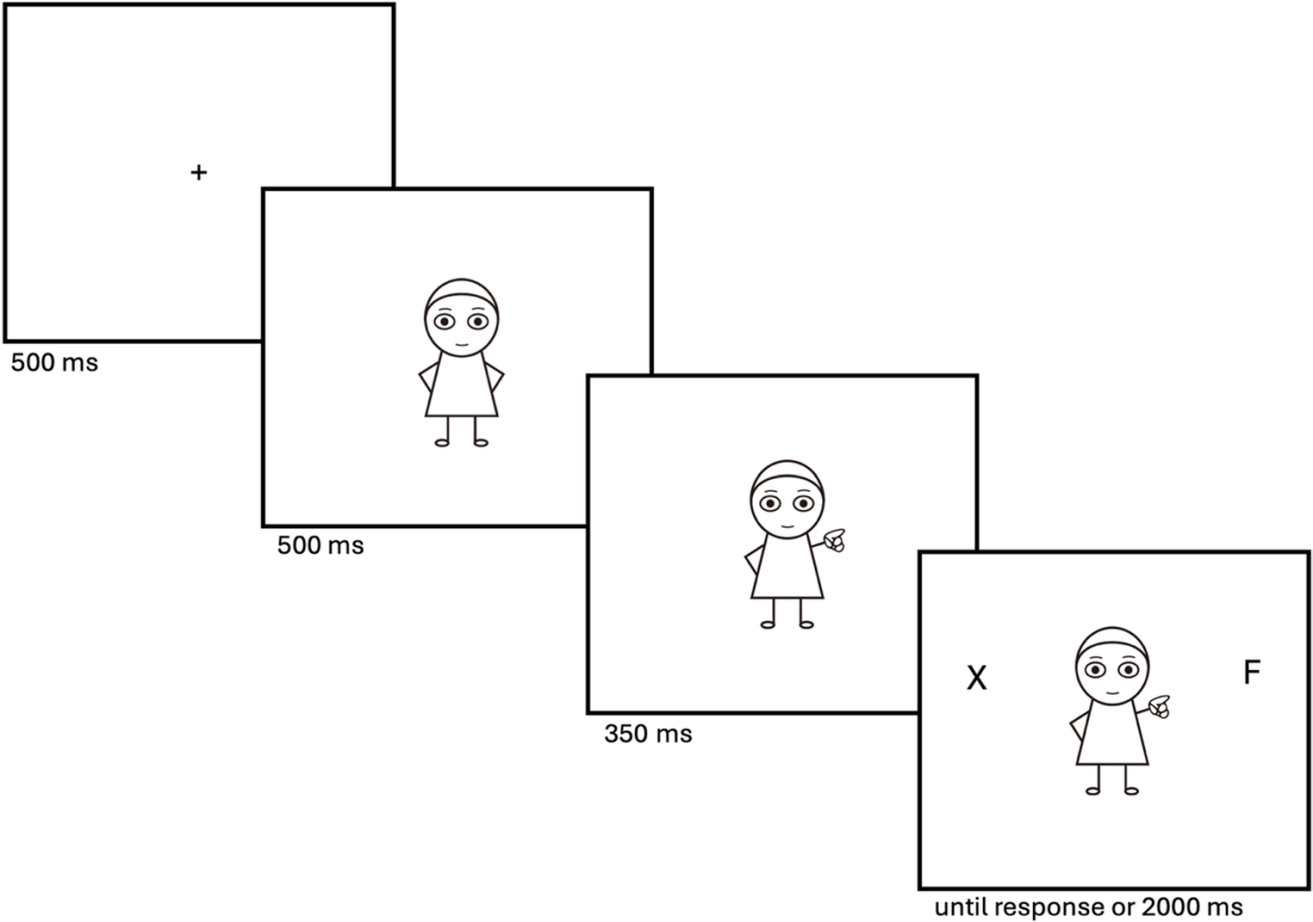
Trial sequence. Note. Trial procedure for experiment 1. This image shows an example of a valid peripheral pointing. Target and response timed out after 2000ms if no response given. Note, the stimuli are not drawn to scale

Participants on Prolific followed the link to the information and consent sheet, which they read and provided their fully informed consent. They were given instructions for the task and asked to respond as accurately and as quickly as possible to the target letter that appeared on-screen. Each trial started with the appearance of a fixation point in the centre of the screen (see Fig. 2). Five hundred milliseconds later, the neutral cartoon figure was presented for 500 ms which contained no directional information. Subsequently, one of the different types of cues was displayed randomly for 350 ms, following which the target letter (“F” or “H”) appeared either on the left or right side of the display. Participants pressed either the ‘f’ or ‘h’ key on the keyboard depending on the identity of the target. There was a block of 16 practice trials to ensure task comprehension. All cue-types were randomly presented in the practice trials. Feedback was provided after each trial; participants were shown a thumbs up emoji on the centre of the display if they responded correctly, and a thumbs down if they answered incorrectly.

There were eight blocks of 32 experimental trials (256 total trials), a total of 64 trials per cue-type presented mixed across blocks. The experimental trials proceeded in the same way as the practice trials, with breaks between blocks providing feedback on accuracy and average response time scores to encourage compliance with the task and motivate their participation. Feedback was presented on the centre of the display. After a total of eight blocks, the participant was thanked for their participation and redirected back to the Prolific website where monetary compensation was given.

#### Analysis

Individual trials were excluded from analysis if an anticipatory keypress was given (within 250ms of target presentation) or the trial timed out without keypress (2000ms after target presentation). A participant’s dataset was excluded if their total error rate was greater than 20%. Incorrect responses and data from practice trials were not analysed.

Data was analysed with a 2 (Validity: valid, invalid) x 4 (Cue type: gaze, flower, peripheral pointing cue, central pointing cue) repeated measures analysis of variance (ANOVA) in JASP (https://jasp-stats.org/).

### Results

Overall, 5.65% of trials of all participants in Experiment 1 were responded to incorrectly and excluded from analysis. Data from two participants was excluded from analysis for having a total error rate greater than 20%. The total number of incorrect trials was 3.64% without these two data sets (*n* = 32). No trial was responded to too quickly, but 0.53% of all trials across participants were excluded for being responded to too slowly (timed out). Overall accuracy was analysed using a 4 (Cue type: gaze, flower, peripheral pointing cue, central pointing cue) x 2 (Validity: valid, invalid) repeated measures analysis of variance (ANOVA). The results showed a main effect of validity *F* (1, 31) = 7.63, *p* = 0.010, η²_p_ = 0.198, showing more accurate responses for valid cue trials (97%) compared to invalid trails (96%). The main effect of cue type was not significant, (F < 1) and neither was the interaction between cue type and validity (F < 1).

Average response times were analysed using a 4 (Cue type: gaze, flower, peripheral pointing cue, central pointing cue) x 2 (Validity: valid, invalid) repeated measures analysis of variance (ANOVA). Sphericity was assumed for the main effect of cue type, X^2^ (5) = 5.686, *p* = 0.338, but not for the cue type x validity interaction, X^2^ (5) = 18.520, *p* = 0.002, so degrees of freedom were corrected using Greenhouse-Geisser estimates of sphericity, ε = 0.721. The results showed there was no significant main effect of Cue type, *F* (3, 93) = 1.089, *p* = 0.358, η²_p_ =.

**Fig. 3:**
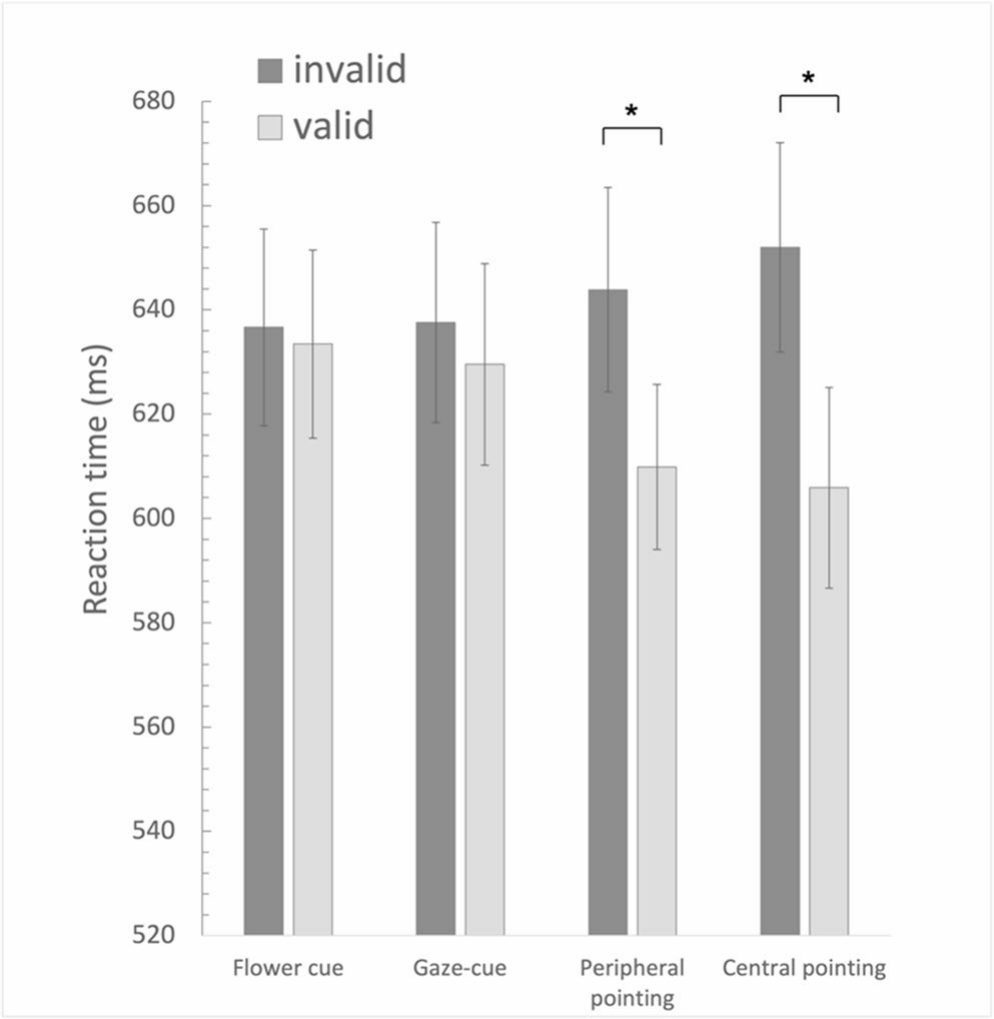
The effect on RT of the four different cue types as a function of validity, presented in mixed blocks in Experiment 1*. *Note: The error bars represent within-subject standard error of the mean. The star denotes a reliable difference between the valid and invalid conditions

0.034. There was a significant main effect of Validity, *F* (1, 31) = 17.599, *p* < 0.001, η²_p_ = 0.362, with significantly faster average response times to valid trials (620 ms) than invalid trials (642 ms). Average response times in each condition of Experiment 1 are shown in Fig. 3.

There was a significant interaction between the effects of cue type and validity on response times, *F* (2.162, 67.031) = 10.164, *p* < 0.001, η²_p_ = 0.247. Looking at Fig. 3, the interaction is likely is due to the larger average response time advantage for valid (compared to invalid) trials in the peripheral and central pointing cue conditions (34 ms vs. 46 ms, respectively) than in the flower and gaze-cue conditions (3 ms vs. 8 ms, respectively). To test this several specific follow up ANOVAs were conducted. To test whether this experiment replicates previous work showing a much stronger validity effect for the pointing-out cue compared to the gaze-cue (Lu & van Zoest, 2023), a follow-up 2 × 2 ANOVA was conducted with Cue type (gaze, peripheral pointing cue) and Validity (valid, invalid) as factors. These results showed no main effect of cue type, F(1, 31) = 1.379, *p* = 0.249, η²_p_ = 0.043, but a main effect of Validity, F(1, 31) = 12.59, *p* = 0.0001, η²_p_ = 0.289, and a significant interaction between cue-type and validity, F(1, 31) = 4.916, *p* = 0.034, η²_p_ = 0.124, showing that the validity effect of the peripheral pointing cue was significantly larger than the validity effect for the gaze-cue. An ANOVA directly comparing the peripheral to the central pointing cue condition, revealed no main effect of Cue type, F(1, 31) < 1, but a main effect of validity, F (1, 31) = 34.17, *p* < 0.01, η²_p_ = 0.524, but no reliable interaction between cue-type and validity, F (1, 31) = 3.256, *p* = 0.081, η²_p_ = 0.009.

Paired samples t-tests were run to test the validity effect for each cue. There was a significant difference between the valid and invalid condition for the peripheral pointing cue (*t* (31) = 4.015, *p* < 0.001) and central pointing cue (*t* (31) = 6.885, *p* < 0.001) cues, but not for the flower (*t* (31) = 0.415, *p* = 0.681) nor the gaze cues, *t* (31) = 0.978, *p* = 0.336.

### Discussion

First, the results replicated the finding that the impact of the pointing hand cues was stronger than gaze cues (Burton et al., 2009; Hermens et al., 2017; Lu & van Zoest, 2023). However, the idea that this benefit may result from closer proximity to the target or greater low-level saliency was not supported. First, performance in the central pointing cue condition was comparable to the peripheral pointing cue condition. In other words, the validity effect was comparable when the hand was extended out as when it was displayed centrally across the body torso, suggesting that spatial proximity did not influence the impact of the pointing cue. Second, even though the flower cue and peripheral pointing cue were matched for low-level visual features, the hand cue biased attention but the flower cue did not. Unlike the flower cue, the pointing cue implies directionality, which appeared critically important in driving the benefit for the pointing cue.

Perhaps somewhat unexpectedly, there was no reliable validity effect for the gaze cue in the present experiment. This was surprising, because the gaze-cue is an extremely robust phenomenon and replicated and confirmed in many different studies and contexts (e.g., Bayliss & Tipper, 2006; Bonmassar et al., 2019; Driver et al., 1999; Friesen et al., 2004; Friesen & Kingstone, 1998; McKay et al., 2021; Ristic et al., 2007; Sato et al., 2007). An absence of a gaze-cue effect in neurotypical adults has been found in studies that manipulated in-group vs. out-group using racial identity (Weisbuch et al., 2017; Zhang et al., 2021), but this tends to be an exception (see also, Dalmaso et al., 2020). A possible explanation for the absence of a gaze-cue effect here, is that the overall context in which the gaze-cue was presented influenced the impact of the cue. Because the four different cue types were presented randomly within blocks, participants may realize the gaze cues were less likely to appear (in 25% of trials) compared to the gesture cues which appeared in 75% of all trials (i.e., flower, peripheral and central pointing cue conditions). Participants’ inferred expectations regarding the lower proportion of the gaze-cues, could have subsequently influenced the susceptibility of this relatively subtle cue.

To investigate the role of overall trial context, the different cue-types were presented separately in different blocks in Experiment 2; validity was still manipulated randomly and unpredictably within each block. It was hypothesized that presenting the different cues in separate blocks would enhance predictability and benefit attentional resources to the relevant features of the cue, and therefore optimize cueing effects (for similar reasoning regarding blocked vs. within design see also, Dalmaso, 2023; Pavan et al., 2011; Zhang et al., 2021). It was predicted that in a blocked set up, the results would be less influenced by intertrial contingencies and enhance the validity effect for the gaze-cue (e.g., Driver et al., 1999; Friesen & Kingstone, 1998).

## Experiment 2

### Method

Experiment 2 was identical in method to Experiment 1, except cues were presented in separate blocks. Using Prolific, 30 new participants (10 male, 20 female) were recruited from the same pool of available participants as Experiment 1. Participant age ranged from 24 to 69 years (*M* = 36.87, *SD* = 12.06). The number of overall trials and trials per cue type was identical to as Experiment 1 (64). There was also an equal number of breaks, splitting each block into two sets of 32 trials. Before each block, participants were shown a new instruction screen telling them which of the cue types they were going to see in that block. Blocks of cue types were presented in a random order for every participant.

Analyses mirrored those performed in Experiment 1, however an additional mixed ANOVA with Experiment as between-subjects was conducted to directly contrast the 4 × 2 repeated measures design in mixed (Experiment 1) and blocked conditions (Experiment 2).

### Results

Overall, 6.85% of trials in Experiment 2 were responded to incorrectly and excluded from analysis. No participants had a total error rate greater than 20%. 0.24% trials were responded to too quickly, 2% of trials across participants were excluded from analysis for being responded to too slowly. Overall accuracy was analysed using a 4 (Cue type: gaze, flower, peripheral pointing cue, central pointing cue) x 2 (Validity: valid, invalid) repeated measures analysis of variance (ANOVA). The results showed a main effect of validity *F* (1, 29) = 29.70, *p* < 0.001, η²_p_ = 0.51, showing more accurate responses for validly cue trials (97%) compared to invalidly trails (94%). The main effect of cue type was not significant, *F* (3, 29) = 1.59, *p* = 0.20, η²_p_ = 0.052, and neither was the interaction between cue type and validity *F* (3, 87) = 1.62, *p* = 0.191, η²_p_ = 0.053.

**Fig. 4:**
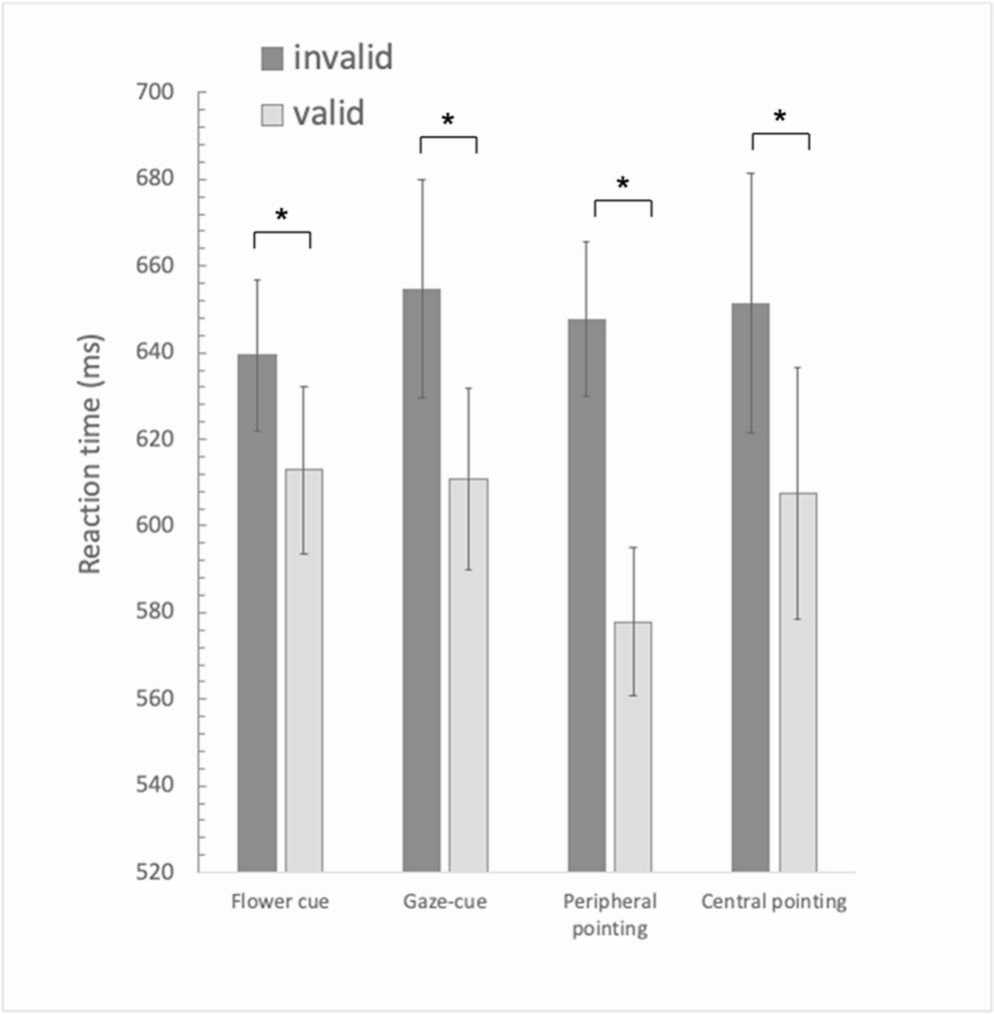
The effect on RT of the four different cue types as a function of validity, presented in separate blocks in Experiment 2*. *Note. The error bars represent within-subject standard error of the mean. The star denotes a reliable difference between the valid and invalid conditions

Average response times were analysed using a 4 (Cue type: gaze, flower, peripheral pointing cue, central pointing cue) x 2 (Validity: valid, invalid) repeated measures ANOVA. Mauchly’s test for the main effect of cue type found it violated the assumption of sphericity, X^2^ (5) = 47.805, *p* < 0.001, so degrees of freedom were corrected using Greenhouse-Geisser estimates of sphericity, ε = 0.576. Sphericity was assumed for the cue type x validity interaction, X^2^ (5) = 7.028, *p* = 0.219. The results showed no significant main effect of Cue type, *F* (1.728, 50.123) = 0.674, *p* = 0.494, η²_p_ = 0.023. However, there was a significant main effect of Validity, *F* (1, 29) = 26.951, *p* < 0.001, η²_p_ = 0.482, with significantly faster average response times to valid trials (633 ms) than invalid trials (692 ms). Additionally, there was a significant interaction between the effects of Cue type and Validity on response times, *F* (3, 87) = 4.773, *p* = 0.004, η²_p_ = 0.141. Average response times in each condition of the blocked Experiment 2 are shown in Fig. 4.

To test whether Experiment 2 replicated Experiment 1 and previous work showing a larger validity effect for the pointing-out cue compared to the gaze-cue (Lu & van Zoest, 2023), a follow-up 2 × 2 ANOVA was conducted with Cue type (gaze, peripheral pointing cue) and Validity (valid, invalid) as factors. These results showed no main effect of cue type, F(1, 29) = 2.19, *p* = 0.149, η²_p_ = 0.034, but a main effect of Validity, F(1, 29) = 26.34, *p* < 0.001, η²_p_ = 0.210. While there was a numerical difference between the validity effect for the gaze-cue (46 ms) and that of for the peripheral pointing cue (72 ms) the interaction between cue type and Validity was just short of being significant, F(1, 29) = 3.97, *p* = 0.064, η²_p_ = 0.009.

Paired samples t-tests were run to compare the invalid to the valid condition for each cue type. The results showed there was a significant difference between valid and invalid cues for each of the cues, for the gaze (*t* (29) = 3.096, *p* = 0.004), peripheral pointing cue (*t* (29) = 6.474, *p* < 0.001), central pointing cue (*t* (29) = 5.203, *p* < 0.001) and the flower cues, *t*(29) = 2.301, *p* = 0.029.

#### Comparing experiments

Additionally, a 4 (Cue type: gaze, flower, peripheral pointing cue, central pointing cue) x 2 (Validity: valid, invalid) x 2 (Experiment: 1, 2; between-subjects factor) mixed ANOVA was conducted on the results of the two experiments together. Mauchly’s test for the interaction of cue type x validity found it violated the assumption of sphericity, X^2^ (5) = 16.100, *p* = 0.007, degrees of freedom were corrected using Greenhouse-Geisser estimates of sphericity, ε = 0.846. There was a two-way interaction between Validity and Experiment, F (1, 60) = 6.45, *p* = 0.014, η²_p_ = 0.004, showing that the overall validity effect was larger in Experiment 2 than 1. There was no three-way interaction between Cue type, Validity, Experiment, *F* (2.537, 152.215) = 1.423, *p* = 0.242, η²_p_ = 0.023.

Separate ANOVAs for each cue-type with Validity as within-subject factor and Experiment as between-subject factor showed that the validity effect for gaze cues, *F* (1, 60) = 5.265, *p* = 0.025, η²_p_ = 0.081, was significantly larger in Experiment 2 (47 ms) compared to Experiment 1 (8 ms). The validity effect was also larger for the peripheral pointing cue in Experiment 2 (72 ms) compared to Experiment 1 (40 ms), *F* (1, 60) = 7.443, *p* = 0.008, η²_p_ = 0.110. Experiment did not significantly alter the validity advantage for flower cues, *F* (1, 60) = 2.904, *p* = 0.094, η²_p_ = 0.046 (3 vs. 27 ms), or central pointing cues, *F* (1, 60) = 0.851, *p* = 0.360, η²_p_ = 0.014 (46 vs. 61 ms).

### Discussion

Experiment 2 had at least two important findings. First, the context in which the trials were presented had an important impact on the impact of the different cues. Second, the result showed a reliable gaze-cue effect in this blocked design; this effect was much larger than when the same cue was presented in the mixed blocks in Experiment 1. The results suggests that the predictable context provided in Experiment 2 increased the impact of the cue. In fact, the predictable situation yielded reliable validity effects for all cue-types.

Experiment 2 provided new insight into the effect of trial context on spatial cues of attention (see also, Langton, 2009). Somewhat surprisingly, the flower cue which did not bear any intentional directional information yielded a statistically significant validity effect. Finding that spatial attention was deployed to the side indicated by the flower may be driven by demand characteristics (Ome, 1962) and a consequence of participants trying to guess what the experiment is trying to measure. Additionally, the directionality of the other cues in the other blocks might have primed participants to similarly interpret the flower as a cue. Evidence for this transfer effect is presented in a study by Ristic and Kingstone (2005), who found specific influence of block order on spatial cuing. They presented participants with an ambiguous cuing stimulus that could be interpreted either as a picture of eyes nearly covered by hat or an automobile – where the eyes can be perceived as wheels of the car depending on the context. The results showed that the automobile was attributed directionality only when participants had first completed the block in which the participants were told the ambiguous cue stimulus contained eyes. The authors argued that top-down control modulates social attention such that attention shifts critically depend on the interpretation of the cueing stimulus. Additionally, once the ambiguous stimulus was perceived as a face, it was extremely difficult to unsee the face and ignore cue direction of this now social cue (Ristic & Kingstone, 2005). Similar priming might explain the results in the flower condition in the present study.^1^

Experiment 3 was designed to find a compromise between the mixed and fully blocked design. In Experiment 3, trials were presented in mixed design, however, this time, the likelihood of the gaze-cue appearing was matched to that of the gesture-cues; instead of one out of four trials in Experiment 1, the gaze-cue was presented in half of all the trials. Relative to Experiment 1, it is predicted that increasing the prevalence of gaze-cue trials would make the gaze-cue more prominent relative to the gesture cues, and that this would subsequently benefit the efficacy of the gaze-cue.

Additionally, in Experiment 3 the time between the cue stimuli and target was manipulated to investigate the time-course of the impact of the different cues (e.g., McKay et al., 2021). By varying the time between the onset of the cue and the onset of the target, participants have more or less time available for processing of the cue which can impact their effectiveness (e.g., Friesen & Kingstone, 1998). Specifically, facilitation at short lead times is thought to reflect reflexive and automatic processing, whereas benefits at longer lead times might involve volitional and strategic processing (e.g., Chanon & Hopfinger, 2011; Driver et al., 1999; Friesen et al., 2004).

## Experiment 3

### Method

Experiment 3 was similar in method to Experiment 1, except as noted below.

#### Participants

For Experiment 3, 97 Participants were recruited from the University of Birmingham, School of Psychology. Like Experiment 1 and 2, participants completed the study on-line, but instead of cash reward they participated for research credits. Participants’ age ranged from 18 to 22 years (M = 19.14, SD = 0.86). The overall number of participants was increased relative to Experiment 1 and 2 to compensate for the reduced number of trials per condition (Von Gunten & Bartholow, 2021). See Design for details.

#### Design

Three cue-types were presented in Experiment 3, specifically (1) the gaze-cue, (2) the peripheral pointing cue, and (3) the flower-cue. Of all trials, 50% were gaze-cue trials, 25% of all trials were peripheral pointing cue and 25% flower-cue trials. The cues were unpredictive of the target location. Stimulus-onset asynchrony (SOA) was varied with 2 levels: short SOA (300 ms) and long SOA (800 ms). Experiment 3 used an SOA that was slightly shorter than the one used in Experiment 1 and 2, based on evidence that the peak of the gaze-cue effect occurs at 300 ms SOA (McKay et al., 2021).

Participants completed 16 practice trials and 256 experimental trials. Experimental trials were divided in 16 blocks of 16 trials each. Feedback was provided after every block. Based on the design, 3 (Cue type: gaze, flower, peripheral pointing cue) x 2 (Validity: valid, invalid) x 2 (SOA: short, long), participants completed 128 gaze-cue trials (32 per each level of validity and soa), 64 pointing cue trials (16 per each level of validity and soa), and 64 flower-cue trials (16 per each level of validity and soa).

### Results

Overall, 4.7% of trials in Experiment 3 were responded to incorrectly and excluded from analysis. Data from three participants were excluded from analysis for having a total error rate greater than 20%. Trials were excluded if they were responded to too quickly (within 250 ms of presentation of the target; 0.06% of trials) or responded to too slowly (> 2000 ms; 0.6% of all trials) and excluded from the final analysis. Overall accuracy was analysed using a 3 (Cue type: gaze, flower, peripheral pointing cue) x 2 (Validity: valid, invalid) x (SOA: short and long) repeated measures ANOVA. The results showed a main effect of validity *F* (1, 93) = 32.48, *p* < 0.001, η²_p_ = 0.259, showing more accurate responses for valid cue trials (97%) compared to invalid trails (96%). The main effect of cue type was not significant, *F* (2, 186) = 1.24, *p* = 0.293 η²_p_ = 0.0513 and neither was the main effect for SOA (F < 1). The interaction between cue type and validity was significant, *F* (2, 186) = 6.97, *p* = 0.001, η²_p_ = 0.07, showing that the validity effect in accuracy was greater for the pointing cue (3.5%) compared to that of the gaze-cue (1.5%) and the flower cue (1.3%). No other contrasts reached significance (all F > 2.67, *p* > 0.073).

Average response times were analysed using a 3 (Cue type: gaze, flower, peripheral pointing cue) x 2 (Validity: valid, invalid) x (SOA: short and long) repeated measures ANOVA. Sphericity was assumed for all contrasts. The results revealed no significant main effect of Cue type, *F* (1, 93) = 0.11, *p* = 0.893, η²_p_ = 0.001. However, there was a significant main effect of Validity, *F* (1, 93) = 72.72, *p* < 0.001, η²_p_ = 0.439, with significantly faster average response times to valid trials (624 ms) than invalid trials (660 ms). There was a significant main effect of SOA, F (1, 93) = 28.48, *p* < 0.001, η²_p_ =0.234., showing responses were faster to the longer SOA (635 ms) compared to the shorter SOA (648 ms). Additionally, there was a significant interaction between the effects of Cue type and Validity on response times, *F* (2, 186) = 34.41, *p* < 0.001, η²_p_ = 0.270.

**Fig. 5:**
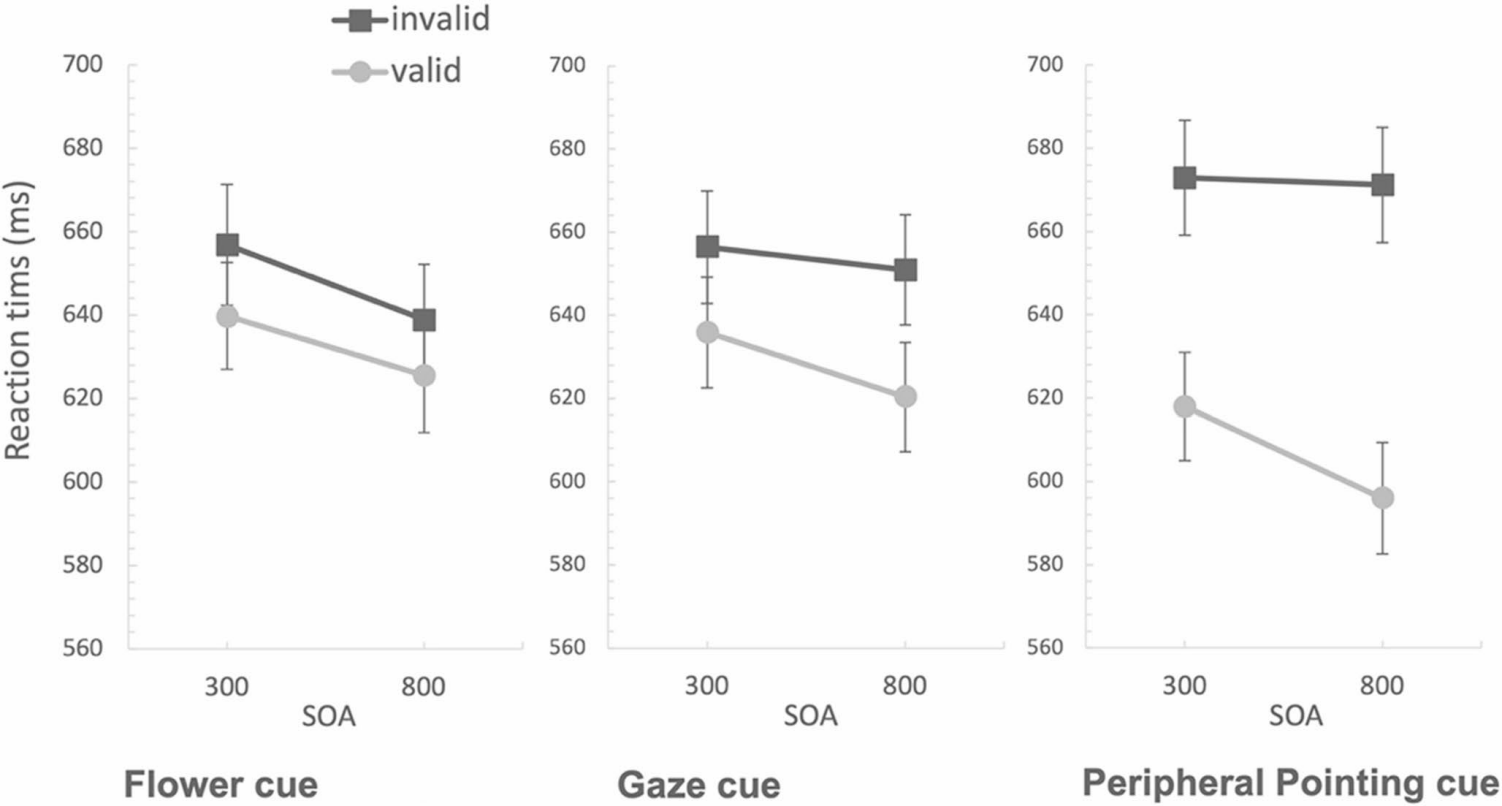
The effect on RT of the three different cue types as a function of validity and SOA in Experiment 3. Note. The error bars represent within-subject standard error of the mean.

A non-significant interaction between SOA and Validity, *F* (2, 186) = 2.42, *p* = 0.123, η²_p_ = 0.025, showed that the validity effect at the long SOA was only numerically larger (40 ms) compared to the validity effect at the short SOA (31 ms). SOA similarly did not interact with any of the other factor factors (all F > 2.3, *p* > 0.10). Average response times in each condition of Experiment 3 are shown in Fig. 5.

To unpack the reliable two-way interaction between Cue type and validity, and test whether Experiment 3 replicated the previous two experiments, a follow up ANOVA with Cue-type (gaze-cue vs. peripheral pointing cue) and Validity (invalid vs. valid) was performed. This analysis showed a reliable interaction, F (1, 93) = 46.23, *p* = 0.009, η²_p_ = 0.332, showing that the cue effect was much larger for the pointing cue (65ms) compared to the gaze-cue (26 ms).

Simple main effects for the validity effect for each for each cue type, showed there was a significant difference between valid and invalid cues for each of the cues, for the gaze cue (*F*(1, 93) = 49.91 *p* < 0.001), the peripheral pointing cue (*F* (1, 93) = 101.49, *p* < 0.001), and the flower cue (*F*(1, 93) = 6.73 *p* = 0.011).

#### Comparing experiments

**Fig. 6:**
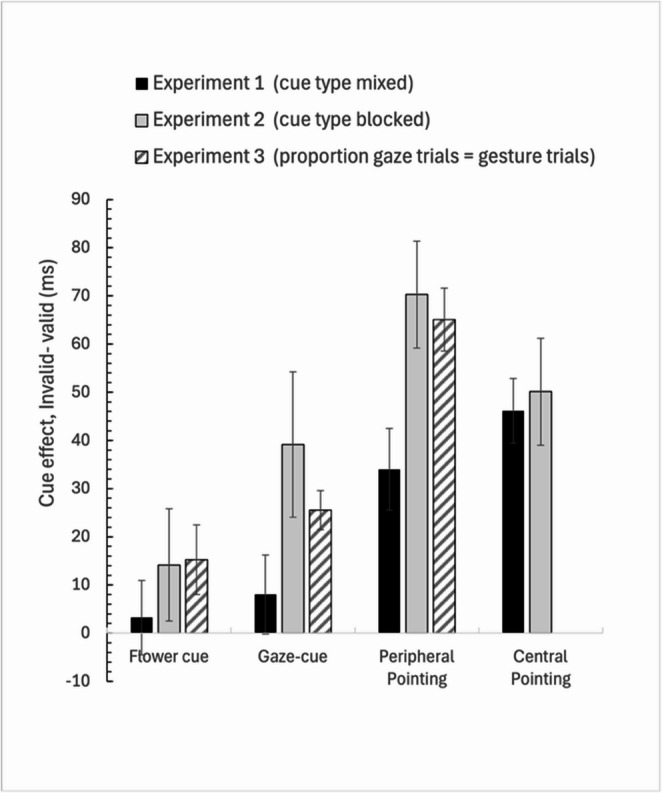
The Validity effect (RT invalid- RT valid) for the different cue types across Experiments 1–3. Note. The error bars represent within-subject standard error of the mean

Additionally, a 3 (Cue type: gaze, flower, peripheral pointing cue) x 3 (Experiment: 1, 2, 3; between-subjects factor) mixed ANOVA was conducted on the cue-effect (RT invalid trials– RT valid trials) of the three experiments together. See Fig. 6. The results showed a main effect of cue-type, F(2, 308) = 22.83, *p* < 0.001, η²_p_ = 0.126, as well as a main effect of Experiment, F(2, 153) = 4.48, *p* = 0.009, η²_p_ = 0.059. There was no interaction between cue-type and Experiment (F < 1) showing that the differences in the validity effect for the different cue-types was similar across experiments. See Fig. 6.

### Discussion

Experiment 3 had at least four main findings. First, the results replicated the results of Experiment 1, showing that the pointing hand cued attention more strongly than the gaze- direction. Second, increasing the total number of gaze-cue trials and matching the overall likelihood of the gaze-cue appearing to the likelihood of the gesture-cues, increased the relative impact of the gaze-cue. In contrast to Experiment 1 where the gaze-cue was presented in one quarter of all trials and the gaze-cue effect not reliable, the validity effect of the gaze-cue was significant in Experiment 3. Third, except for a simple main effect of SOA, SOA did not modulate performance. This suggests that in this experiment, the time-course of the validity effect was comparable for the different types of cues. It may be that the range of tested SOAs (300 and 800 ms) was not sufficiently extensive to capture time-course differences in the processing of the different cues. In the future, a more thorough investigation using a broader distribution of stimulus onset asynchronies (SOAs) would help strengthen the conclusions. Fourth, like the results of Experiment 2, the results of Experiment 3 revealed a reliable influence of the flower cue on attentional performance.

Across experiments, the results showed that although the experiment influenced the overall effect size of the cue on attentional performance, the critical interaction between cue type - specifically, the greater validity effect for the peripheral pointing cue relative to the gaze cue - remained stable across experiments. This pattern suggests that the pointing cue is more robust than the gaze cue, and that this advantage generalizes across different settings regardless of variations in trial context and expectations about stimulus presentation.

## General discussion

The results of the present study showed that when looking at embodied cues, the pointing cue is a more effective cue than the gaze-cue. The effectiveness of the pointing cue could not be explained by low-level salience, as the flower cue that was matched for low-level saliency did not cue spatial attention to the same degree. Moreover, the impact of the pointing cue was equally large when the hand was extended out as when it was displayed centrally across the body torso, suggesting that spatial proximity did not influence the impact of the pointing cue. The present results suggest that the pointing index finger is unique in directing attention (see also, Ariga & Watanabe, 2009). Furthermore, the results showed that trial context greatly affected the overall effectiveness of the cues but did not change the interactions between the different cue-types. Thus, although spatial cueing is influenced by expectations and is to some extent malleable, the pointing cue emerges as the strongest spatial cue.

What then explains the large differences in effectiveness of the pointing cue compared to the gaze-cue, if it is not saliency and low-level features? One hypothesis to consider in the future, is the role of implied intentionality and effort that is different between pointing- and gaze-cues. Pointing movements are generally made voluntarily and intentionally; they require coordination and are often directed with the aim to inform other people. In contrast, most eye movements are triggered unintentionally and directed with the main aim to bring relevant information onto the fovea for high-acuity vision. Not every shift in eye-gaze is a signal intended to initiate joint attention. While shifts in gaze often occur reflexively, pointing movements may be intuitively perceived as more intentional and directive; that is, actors would not normally point inadvertently, without meaning to make a ‘point’. This difference in implied intentionality of the signal could be what is reflected in the increased effectiveness of the pointing cues over the gaze-cue.

Reasoning further, this intentionality hypothesis predicts that the impact of the embodied pointing cue might be more powerful in comparison to a symbolic arrow cue. While symbolic arrows may involve implicit intentionality, embodied pointing cues provide a more clearly motivated and intentional cue of directionality. While pointing cues are intuitively similar to arrow cues, to the best of our knowledge no study has directly compared the pointing cue and the arrow cue. Previous work comparing the symbolic arrow cue to the gaze-cue show that biases in response to the symbolic arrow cue are very similar to the gaze-cue (e.g., Chacón-Candia et al., 2023; Tipples, 2002; however see, Schmitz et al., 2024). Based on the present results, one would predict an advantage of the pointing cues over the symbolic arrows cues.

The gaze-cue is often referred to or classified as a social cue, in contrast to symbolic arrow cues that are considered non-social (e.g., Hayward & Ristic, 2015). The social label given to gaze-cues is based on the idea that gaze-direction involves mental attribution, and this theory of mind and higher cognitive processing is what makes them fundamentally social (e.g., Morgan et al., 2018; Nuku & Bekkering, 2008; Teufel et al., 2010; Wiese et al., 2013). However, while evidence suggests mental attribution can modulate the gaze-cue effect (e.g., Morgan et al., 2018), mental attribution alone may not be sufficient to bias attention (Cole et al., 2015; Kingstone et al., 2019). For example, Kingstone and colleagues (2019) investigated gaze-cuing in a situation where they removed the asymmetry in the gaze stimuli, ensuring that mental attribution was essential to infer gaze position. In this study, they presented short videos of a masked person looking straight ahead and then turning to side before the appearance of the target. The critical part was when the person was wearing two masks, one on the face and on the back of the head, such that when the person turned to look to the side, the final view before the presentation of the target, was of two masks viewed from the side and perfectly symmetrical. The target subsequently appeared on the side the person turned to (validly cued), or the opposite side (invalidly cued). Observers could infer the cued position only when they had attributed mental capacity to the face and remembered their initial position. However, the results revealed no evidence for a validity effect, suggesting that people did not use this information. A spatial cuing effect was found only when the cue was asymmetric and mental attribution not required (Kingstone et al., 2019).

The results of Kingstone and colleagues point to the importance of an asymmetry in the features of the cues. This idea is related the flower findings in the present study; the asymmetric flower cue (even though bearing no intuitive directional significance) reliably biased spatial attention in both Experiment 2 and 3. It is difficult to argue that mental attribution was involved in the flower cue condition. These results support the idea that the presence of asymmetry in the cue stimuli may drive spatial cueing rather than mental attribution and theory of mind. Moreover, whether eye gaze is used as a source of information in real-life depends on social circumstances. Evidence suggests that people often avoid looking directly at other people in social situations, yet are much more open to look at faces directly when they are not observed or when there is no potential for social interaction (Laidlaw et al., 2011, 2016). Gaze-cueing does not rely on direct social interactions (see Laidlaw et al., 2011), and seems equally effective if presented in photos or schematic pictures (Dalmaso et al., 2025), cartoons or animals (Quadflieg et al., 2004). These findings question whether the use of the ‘social’ label for gaze-cueing is useful in describing or classifying the cue in typical attentional paradigms. All the same, while mental attribution may not be necessary to bias attention to various cues (Kingstone et al., 2019) this does not exclude the idea that higher social and cognitive processes affect the processing of the cued stimuli (for a review see, Dalmaso et al., 2020).

To conclude, the results of this study show that the pointing hand is a very effective cue of visual attention and more powerful than the gaze-cue. The impact of the pointing cue could not be explained by low-level salience, and the impact of the pointing cue was equally large for the peripheral and central pointing cue, suggesting that spatial proximity did not influence the impact of the pointing cue. It is concluded that the pointing hand is unique in directing attention, but that overall context and expectations shape the efficacy of cues in general. While the label ‘social’ given to embodied cues like the gaze- and pointing-cue can be useful in descriptive sense, our results suggests that the predictive validity of this categorization is questionable.

## Data Availability

Stimuli materials and data are available on: https://osf.io/ftrmc/.

## Footnotes

1 To further explore this idea, the validity effect for the flower cue was analysed separately for participants who completed the flower block before the peripheral pointing cue and compared this to participants who completed the peripheral pointing cue before the flower cue. If the peripheral pointing cues primed people to interpret the flower cue in a comparable way, the validity effect for the flower should only arise when this block was completed after the pointing cue as compared to before the pointing cue block. An independent samples T-test on the validity effect for the flower cue with order as between-subject factor revealed no reliable difference between orders (t(25) = 0.69, *p* = 0.5). While the validity effect for was numerically larger for participants who completed the flower cue block after (31 ms, *n* = 17) compared to first (12 ms, n= 10), neither were significant from zero [t(16) = 1.89, *p* = 0.077 and t(9) = 0.61, *p* = 0.558].
